# Watching sports events and residents’ subjective well-being: evidence from the CGSS and the potential roles of health and social capital

**DOI:** 10.3389/fpsyg.2026.1775253

**Published:** 2026-02-04

**Authors:** Xuemeng Wei, Shutong Zhao

**Affiliations:** 1 College of Physical Education, West Anhui University, Lu’an, China; 2 School of Economics and Management, Shanghai University of Sport, Shanghai, China

**Keywords:** health capital, mental health, social capital, sport spectatorship, subjective well-being, watching sports events

## Abstract

**Introduction:**

Investigating the association between watching sports events and residents’ subjective well-being and the potential mediating mechanisms advances understanding of the positive psychological correlates of sports spectatorship and provides a basis for efforts to support well-being and promote social harmony.

**Methods:**

Based on 8 years of the Chinese General Social Survey (CGSS) data spanning 2010–2023, we employed OLS regressions with province and survey-year dummy variables, Ordered Probit and Ordered Logit regressions, and propensity score matching (PSM) to examine the association between watching sports events and subjective well-being. We also tested whether health capital and social capital statistically accounted for part of this association.

**Results:**

Watching sports events was positively associated with subjective well-being, and the association remained after heterogeneity analyses and selection-adjustment checks. The association was strongest in the West, followed by the Central and the East. It was significant for men but not for women. By education, the association was largest among high-school-educated residents, smaller but significant among those with college and above, and not significant among those with primary or lower-secondary education. Mediation analyses were consistent with social capital (social class, social trust, and social support) and physical health, statistically accounting for part of the association between watching sports events and subjective well-being; by contrast, mental health did not emerge as a primary statistical pathway.

**Conclusion:**

These results advance knowledge of how watching sports events relates to well-being and offer policy-relevant insights for promoting happiness through accessible, low-cost leisure engagement, while also acknowledging potential downsides of excessive viewing.

## 1 Introduction

For decades, meeting citizens’ aspirations for a good life has been a key goal of China’s state governance. However, economic expansion does not automatically translate into higher life happiness, making the determinants of well-being an important policy and research concern. In academic research, happiness is commonly quantified using subjective well-being (SWB), defined as individuals’ overall evaluation of their quality of life. SWB captures both cognitive judgments (life satisfaction) and affective experiences (positive and negative affect) (Diener, 1984; Diener et al., 2009), and it has become a widely used indicator for evaluating social progress and public policy (Easterlin, 1974).

Among the many determinants of SWB, everyday leisure behaviors are particularly policy-relevant because they are modifiable, scalable, and embedded in daily life. Sport is one such behavior: it is widely promoted as a means to enhance health, social connection, and quality of life, yet it also includes “passive” forms of engagement that may reach broader segments of the population than active participation.

Sports participation comprises direct forms (exercise, physical activities) and indirect forms (sport spectatorship, consumption). While the well-being benefits of physical activity are well established (van Woudenberg et al., 2020; Feng et al., 2024), evidence on sport spectatorship remains comparatively limited and mixed. Prior research has disproportionately examined (i) SWB “feel-good” effects linked to mega-event hosting or national-team success (e.g., the Olympic Games and major football tournaments) and (ii) sport- or event-specific settings, particularly football and World Cup contexts, in which identification may yield short-lived SWB gains but can also trigger marked stress responses (Kavetsos and Szymanski, 2010; Xu and Sato, 2025). This emphasis on episodic or sport-specific settings leaves open a more everyday question: whether routine watching, as a repeatable leisure behavior, is reliably associated with residents’ SWB.

Routine cross-sport watching via television or online media is low-cost and widely accessible, yet it has been studied mainly outside China (e.g., the UK and Japan), leaving limited nationally generalizable evidence for China (Keyes et al., 2023). Descriptive indicators nonetheless suggest that everyday sports viewing is common and feasible in China. Consumer surveys report large audiences for mainstream sports (e.g., basketball and soccer) (Shafer and Heng, 2023). National digital-infrastructure statistics also document widespread access to online video and live-streaming services, making routine viewing possible through both television and mobile apps (China Internet Network Information, 2025). In addition, the marginal cost of routine viewing is often low because sports content is widely available via free-to-air broadcasting and major platforms that provide free access alongside low-priced premium tiers (Tencent Holdings, 2025). Existing China-based studies, however, often draw on specific subgroups or non-national samples rather than repeated nationally representative waves (Guo et al., 2024). Accordingly, we examine whether everyday sports-event watching—independent of any single mega-event or sport—is associated with residents’ SWB in China and through which channels. This focus on routine watching is distinct from mega-event spectatorship because routine viewing is repeatable and embedded in daily leisure, whereas mega-events are episodic and may be driven by event-specific shocks such as temporary national pride or stress, which limits generalizability and policy relevance. To address this gap, we draw on eight waves of nationally representative data from the Chinese General Social Survey (CGSS, 2010–2023). We estimate OLS regressions with province and survey-year dummy variables and implement a series of robustness and selection-adjustment strategies (including propensity score matching) to mitigate concerns about confounding and self-selection. We further examine heterogeneity by region, gender, and educational attainment, and test whether health capital and social capital statistically account for part of the association between watching and SWB.

This study contributes in three ways. First, it provides large-scale evidence on passive sport engagement and SWB in a major emerging economy. Second, it articulates a theory-informed framework that links spectatorship to SWB through health capital and social capital, and empirically examines whether these factors statistically account for part of the association. Third, by combining province and survey-year dummy variables with multiple robustness checks, the analysis provides additional robustness evidence on the relationship between watching sports events and residents’ subjective well-being.

### 1.1 Watching sports events and subjective well-being

Building on the research gap identified above, we focus on routine sports-event watching as an everyday form of passive sport engagement and develop a psychologically grounded explanation for why it may relate to SWB. Subjective well-being (SWB) is a key metric of psychological health and social well-being, capturing both overall life satisfaction and positive emotional experiences in daily life (Diener, 1984; Steptoe et al., 2015). As sports broadcasting and media formats have diversified, watching sports events has become a prevalent form of leisure, raising interest in how it relates to SWB. From a psychological perspective, routine sports spectatorship may be linked to SWB through three complementary mechanisms: (i) emotion regulation and recovery experiences (e.g., mood management and stress relief), whereby engaging sports content helps regulate momentary affect and may spill over into global well-being appraisals (Gross, 1998); (ii) social identity processes, whereby identifying with a team or fan community provides belonging, pride, and self-esteem (Tajfel and Turner, 2004); and (iii) basic psychological need satisfaction (relatedness, competence, autonomy), as spectators experience shared connection, vicarious achievement, and intrinsically chosen leisure engagement (Wann, 2006; Funk et al., 2012). Importantly, sports spectatorship does not require bodily exertion and therefore should not be treated as a proxy for physical activity. Instead, spectatorship can function as an independent psychological stimulus embedded in shared rituals (co-viewing, discussion, and community ties). At the same time, spectatorship may involve countervailing mechanisms, including prolonged sedentary time, stress reactivity during high-stakes matches, and sleep disruption due to late-night viewing, which may attenuate or reverse benefits for some viewers.

Prior studies suggest that watching sports events provides immediate affective benefits and may relate to well-being via social interaction and identification. Drawing on data from five Chinese provinces/municipalities, Guo et al. (2024) reported a significant positive association between watching sports events and subjective well-being, with patterns consistent with an indirect association involving social interaction and emotional experience; however, given the cross-sectional design, these pathways should be interpreted as statistical associations rather than causal mechanisms. In a UK-based empirical study, Ramchandani et al. (2022) similarly found that spectators at major sports events report higher life satisfaction, subjective well-being, and self-worth. Experimental evidence from Lin et al. (2023) indicated that individuals who watched sports television content for 3 weeks reported higher well-being than those who did not, with the effect especially pronounced among those highly engaged with sports, suggesting that the match between viewing and personal interest matters. From a neuroscience perspective, Kinoshita et al. (2024a) used neuroimaging experiments to demonstrate that watching sports events activates brain regions associated with well-being, and that viewing popular sports elicits stronger feelings of happiness than less-followed sports. Overall, existing evidence points to a generally positive but likely modest and context-dependent association between routine sports watching and SWB, especially when spectatorship is socially shared and identity-relevant. Although some studies suggest that specific mega-events (e.g., the Olympic Games) exert limited effects on host-country happiness (Bodin et al., 2021), routine sports-event watching is repeatable and broadly accessible and may therefore be positively associated with residents’ subjective well-being. Accordingly, we propose the hypothesis:

*H1*: Watching sports events is positively associated with residents’ subjective well-being.

### 1.2 Watching sports events, health capital, and subjective well-being

Beyond these immediate affective and identity-related benefits, spectatorship may also be linked to SWB through health-related resources, consistent with the view that well-being partly reflects mental and physical health endowments. The concept of health capital was introduced by economist Michael Grossman in 1972 to explain how individuals regard health as a “capital stock” that can be augmented through investment and depreciated over time (Grossman, 1972). In accounting for health inequalities, researchers note that both physical and mental health reflect an individual’s endowment of health capital (Pinxten and Lievens, 2014). In the present context, we treat mental health as a proximal affective and stress-related state closely tied to SWB, while physical health reflects vitality and functional capacity that support daily positive experiences and life satisfaction.

Mental health is one of the key determinants of residents’ subjective well-being (Mathentamo et al., 2024). Watching sports events is often accompanied by the generation of positive emotions, strengthened group belonging, and the release of tension and stress, all of which can improve individuals’ mental health (Wann, 2006; Inoue et al., 2017a). From an emotion-regulation and social-identity lens, spectatorship may contribute to lower distress and better psychological functioning by providing mood repair, meaning, and belonging (Wann, 2006). Scholars have noted that sporting events provide a distinctive social and emotional experience; the positive affect and social interaction derived during spectating help alleviate anxiety and loneliness and enhance psychological well-being (Kavetsos and Szymanski, 2010). Thus, any positive association between watching and SWB may be partly accounted for by better mental health. Accordingly, we propose the following hypothesis:

*H2a*: The positive association between watching sports events and residents’ subjective well-being is partly explained by better mental health.

Physical health serves as a necessary foundation for happiness and a critical guarantee of life quality (Wang et al., 2022). Prior studies indicate that participation in sports contributes to better physical health, which in turn enhances well-being (Downward and Dawson, 2016). While watching sports events is an indirect form of sport engagement (rather than active participation), it can still prompt exercise intentions and heighten fitness awareness, thereby indirectly improving physical health (Funk et al., 2012). In addition, watching sports events is often associated with healthier lifestyle choices (e.g., joining community sports activities, greater sports consumption), which may further support physical health (Kim and James, 2019). At the same time, spectatorship can also be sedentary; therefore, any positive association via physical health is theoretically plausible but may be weaker and contingent on whether watching stimulates subsequent active behaviors. Accordingly, we propose the following hypothesis:

*H2b*: The positive association between watching sports events and residents’ subjective well-being is partly explained by better physical health.

In parallel to health-related pathways, spectatorship is also a social behavior that may generate relational resources and broader social benefits. We therefore consider social capital as a second set of mechanisms linking watching to SWB.

### 1.3 Watching sports events, social capital, and subjective well-being

Social capital, as a vital societal resource, plays a pivotal role in enhancing individuals’ quality of life and well-being. It encompasses not only upward shifts in social status but also increases in social trust and social support (Putnam, 2000). Existing studies indicate that social capital can substantially promote residents’ subjective well-being by facilitating access to resources, strengthening group belonging, and improving the quality of social interactions (Kawachi et al., 1997). Psychologically, these benefits map onto belongingness and social identity processes: shared fandom can strengthen perceived connectedness, mutual trust, and felt support, all of which are well-established correlates of SWB. Within this framework, watching sports events, as a form of socially embedded leisure, may indirectly be associated with SWB by reinforcing individuals’ social capital.

Social class is an essential component of social capital (Carmo and Nunes, 2013); improvements in social class can enhance individuals’ social standing and access to entitlements, thereby positively influencing well-being (Carmo and Nunes, 2013). As a key domain of social interaction, sport is regarded as a mechanism for social mobility, helping individuals establish positive community ties and, indirectly, accumulate social capital (Harris and Parker, 2009). Prior research indicates that sports consumption and participation may promote the accumulation of sociocultural and symbolic capital, which in turn strengthens individuals’ perceived social class (Bourdieu, 1986). Sporting events often serve as important channels for mobility and class identification (Rungo and Sánchez-Santos, 2022). Through watching sports events, residents obtain more opportunities for social interaction and cultural identification; this symbolic improvement in social status may contribute to greater subjective well-being (Wilson, 2002). Accordingly, we propose the following hypothesis:

*H3a*: The positive association between watching sports events and residents’ subjective well-being is partly explained by higher perceived social class.

Social trust is a core component of social capital and a key determinant of well-being (Helliwell et al., 2016). As prototypically collective activities (Yoshida et al., 2023), sporting events can generate shared emotional experiences and a sense of identification among spectators, thereby strengthening social trust (Putnam, 2000; Kawachi et al., 1997). Empirical studies indicate that participating in or watching sports can foster cooperation and reciprocal behaviors across groups, which in turn increases trust at both interpersonal and societal levels (Wann et al., 2008). In this context, watching sports events may foster shared contexts and value congruence, which helps increase social trust and, in turn, is associated with higher subjective well-being. Accordingly, we propose the following hypothesis:

*H3b*: The positive association between watching sports events and residents’ subjective well-being is partly explained by higher social trust.

Social support is another core dimension of social capital, referring to the emotional, informational, and instrumental assistance individuals obtain within social networks (Lin, 2002). Prior research indicates that social support significantly improves psychological well-being and life satisfaction (Cohen and Wills, 1985). Watching sports events is often undertaken with family members, friends, or colleagues; such shared experiences strengthen interpersonal relationships and emotional bonds (Guo et al., 2024; Wann, 2006). In this process, residents may perceive greater emotional support and social recognition, which in turn may be associated with higher subjective well-being (Lin, 2022). Accordingly, we propose the following hypothesis:

*H3c*: The positive association between watching sports events and residents’ subjective well-being is partly explained by stronger social support.

**Figure 1:**
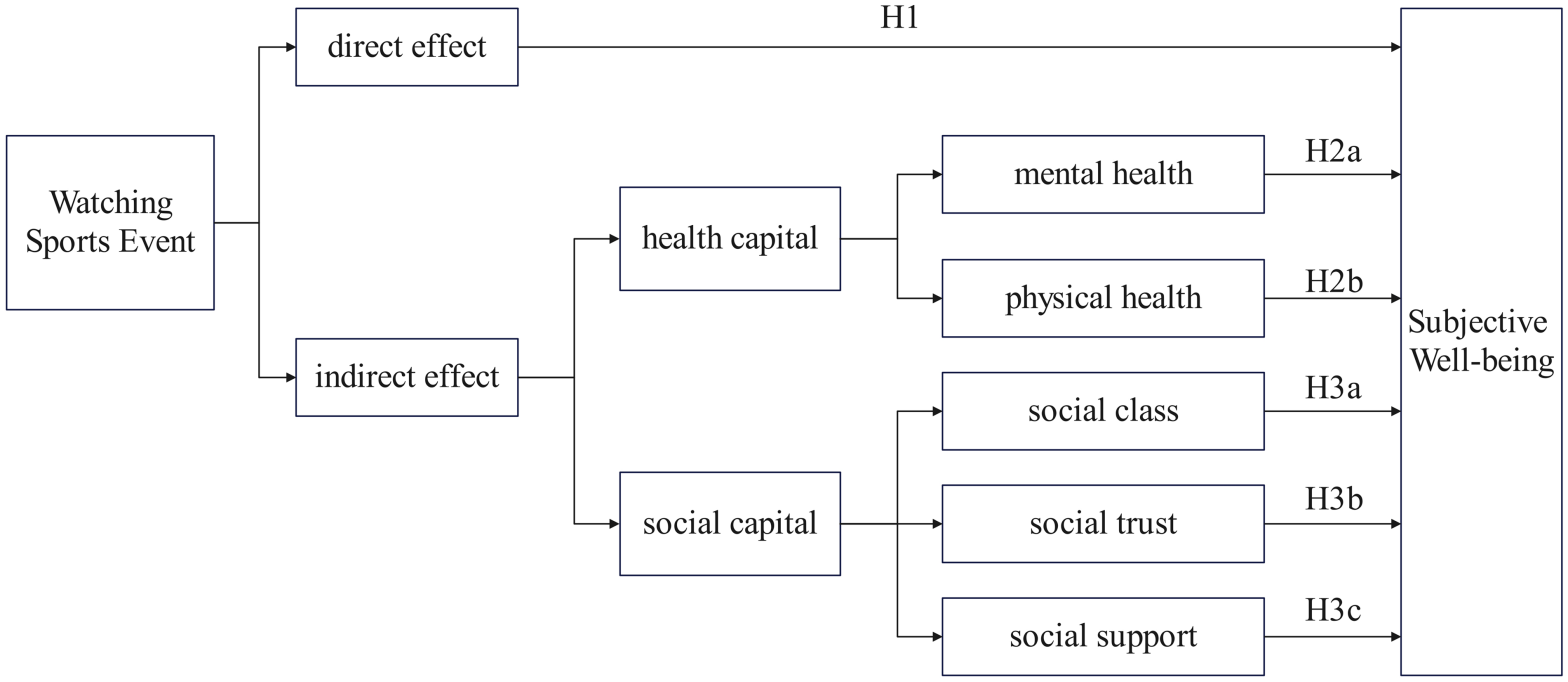
Theoretical model.

Overall, we test whether health-related and social-capital-related variables statistically account for part of the association between watching sports events and SWB; these analyses do not establish causal mediation given the repeated cross-sectional nature of the data. Figure 1 summarizes the conceptual model and positions heterogeneity as a set of boundary conditions under which these associations may be stronger or weaker. This study develops a theoretical model with health capital and social capital as mediating factors to clarify how watching sports events may relate to residents’ subjective well-being, as shown in Figure 1.

### 1.4 Heterogeneous effects of watching sports events on subjective well-being

Residents’ subjective well-being is closely associated with sociodemographic characteristics such as gender (Inglehart, 2002), age (Koo et al., 2004), place of residence (Navarro et al., 2020), educational attainment (Jin et al., 2020), and occupation (Christiansen et al., 1999). The association between subjective well-being and watching sports events is not uniform across all groups but exhibits pronounced heterogeneity. Owing to differences in economic conditions, social environments, and value orientations, residents from different regions, genders, and educational levels may show varying associations between spectating behavior and well-being (Diener et al., 2018). From a psychological standpoint, heterogeneity is also expected because emotion-regulation motives, identity investment, and opportunities for social sharing differ across contexts and social groups, shaping when and for whom spectatorship is most strongly linked to SWB.

Imbalances in regional economic development, cultural backgrounds, and the allocation of sports resources may lead to differential associations between spectating behavior and residents’ well-being. Prior research suggests that regional disparities are a key factor in explaining variations in subjective well-being (Easterlin et al., 2012). In China, residents in the eastern region may obtain greater benefits from watching sports events due to a more mature industrial structure and a richer supply of sports resources, whereas residents in the central and western regions may experience stronger well-being associations owing to the relative scarcity of sporting events and the accompanying social connectedness. These patterns may reflect variation in both access and the social meaning of spectatorship, including (a) accessibility and viewing quality and (b) the extent to which viewing is embedded in collective identity and social interaction.

Does gender shape the well-being association of watching sports events? Gender differences have long been a central topic in research on subjective well-being (Inglehart, 2002; Batz-Barbarich et al., 2018). A review of existing literature indicates marked differences between men and women in sports consumption and psychological responses (Ruseski et al., 2014). Men tend to seek recognition and social status through sporting events, whereas women may rely more on social interaction and emotional fulfillment (Gantz and Wenner, 1991). Accordingly, gendered differences in identity motives and social–emotional benefits suggest that the strength of the association between watching and SWB may differ by gender.

Educational attainment shapes individuals’ values, lifestyles, and abilities to access social resources, thereby playing a crucial role in the formation of subjective well-being (Oreopoulos and Salvanes, 2011). Highly educated groups typically possess richer social capital and greater purchasing power (Ferrer Esteban and Mediavilla, 2017), enabling them to obtain more psychological satisfaction and social identification through watching sports events; by contrast, individuals with lower educational attainment may face economic constraints and limited access to viewing channels, resulting in a comparatively modest well-being association. Education may also influence cognitive appraisal and leisure autonomy, which can strengthen or weaken how spectatorship relates to SWB. Based on this, we propose the following hypotheses:

*H4a*: The association between watching sports events and residents’ subjective well-being exhibits regional heterogeneity.

*H4b*: The association between watching sports events and residents’ subjective well-being differs by gender.

*H4c*: The association between watching sports events and residents’ subjective well-being differs by educational attainment.

## 2 Materials and methods

### 2.1 Sampling

The data used in this study were drawn primarily from the Chinese General Social Survey (CGSS). Initiated in 2003, the CGSS is a nationally representative, authoritative, comprehensive, and continuous academic survey in China that spans politics, economics, culture, and society, and it has been widely employed in social science research. The key explanatory variable in this study, watching sports events, is not collected in all CGSS waves. Therefore, we selected all survey years in which this item is available and measured in a comparable manner, resulting in eight waves: 2010, 2012, 2013, 2015, 2017, 2018, 2021, and 2023. This approach maximizes sample size and statistical power while ensuring measurement comparability of the focal variable across waves. To accurately and comprehensively reflect the empirical context, we excluded observations with “do not know” or “refuse to answer” responses, as well as outliers and anomalous values, and applied a 1% winsorization to all continuous variables. The final sample comprises 53,967 valid observations, providing a strong basis for inference and analytical value. Descriptive characteristics of the sample are reported in Table 1. To address potential heterogeneity across survey years (e.g., common shocks, changes in social context, or survey timing), all regression models include survey-year dummy variables (along with province dummies) to absorb time-specific factors shared by respondents within each wave. All analyses were conducted using Stata 18.0.

**Table 1:** Demographic characteristics of the sample (*N* = 53,967).

Characteristics	Category	Frequency	Percentage %
Year	2010	2,755	5.10
	2012	9,009	16.69
	2013	8,287	15.36
	2015	8,001	14.83
	2017	9,168	16.99
	2018	9,239	17.12
	2021	3,230	5.99
	2023	4,278	7.93
Gender	Male	28,370	52.27
	Female	25,597	47.43
Age	Youth (18–34 years old)	9,374	17.37
	Middle-aged adults (35–64 years old)	32,529	60.28
	Elderly (≥65 years old)	12,064	22.35
Marital status	Married	43,054	79.78
	Unmarried and other	10,913	20.22
Educational attainment	Primary school or below	18,195	33.72
	Junior high	15,900	29.46
	High school	10,249	18.99
	University or above	9,623	17.83
Household registration	Rural	28,364	52.56
	Urban	25,603	47.44
Region of residence	Eastern Region	20,735	38.42
	Central region	12,498	23.16
	Western region	13,775	25.52
	Northeast Region	6,959	12.89

### 2.2 Measurements

#### 2.2.1 Dependent variable

Subjective well-being served as the dependent variable. It was measured using the CGSS item, “Overall, how happy do you feel about your life?” Responses range from 1 to 5, where 1 = “very unhappy,” 2 = “relatively unhappy,” 3 = “neither happy nor unhappy,” 4 = “relatively happy,” and 5 = “very happy.” Higher scores indicate greater subjective well-being. This item captures respondents’ global evaluative appraisal of their life happiness at the time of the survey. Given data constraints and the need for consistency across the eight CGSS waves, we use this single-item global measure as our operationalization of SWB. We acknowledge that SWB can be defined more broadly to include both cognitive judgments and affective experiences; however, the CGSS does not provide a consistent multi-item battery covering all components across waves. Because a single-item measure does not allow internal-consistency reliability assessment and may contain measurement error, our estimates should be interpreted as associations that may be attenuated toward zero. We further discuss this conceptual and measurement limitation in the Limitations section.

#### 2.2.2 Independent variable

Watching sports events served as the independent variable. It was measured using the CGSS item: “During the past year, in your leisure time, did you often watch sports events?” Response options ranged from 1 to 5, coded as 5 = “never,” 4 = “several times a year or less,” 3 = “several times a month,” 2 = “several times a week,” and 1 = “every day.” In this study, “watching sports events” is operationalized as any self-reported engagement in sports-event viewing during leisure time over the past year (i.e., at least occasionally), rather than a measure of habitual, high-intensity fandom or identification. We then constructed a binary indicator by recoding “every day,” “several times a week,” “several times a month,” and “several times a year or less” as 1 (watching sports events), and “never” as 0. This dichotomization is used as the main specification because the higher-frequency categories have relatively small cell counts, which may yield unstable estimates if modeled separately; the original frequency scale is additionally examined in robustness checks.

#### 2.2.3 Mediator variables

Consistent with the preceding literature review, health capital comprises physical and mental health. In this study, we operationalize “mental health” as recent psychological distress/negative affect, measured using the CGSS item: “During the past 4 weeks, how frequently have you felt depressed or downhearted?” Responses are on a five-point scale—“always,” “often,” “sometimes,” “seldom,” and “never” coded from 1 to 5, respectively, such that higher scores indicate less frequent depressive feelings and thus better recent psychological well-being. We note that this single item captures only one salient facet of mental health (depressive affect) rather than a comprehensive assessment (e.g., anxiety, positive affect, clinical symptoms, or broader functioning).

Physical health was measured using the CGSS item: “How would you rate your current physical health status?” Responses are on a five-point scale—“very unhealthy,” “relatively unhealthy,” “average,” “relatively healthy,” and “very healthy”—coded 1–5, respectively. Higher scores indicate better physical health.

Social capital comprised three dimensions: social class, social trust, and social support. For the mediating variable of social class, we used the CGSS item, “Where would you place yourself on the social ladder at present?” Responses range from 1 (lowest) to 10 (highest) and were coded 1–10 accordingly, with higher values indicating a higher perceived social class.

Social trust was treated as a mediating variable. It was measured using the CGSS item: “Overall, do you agree that, in this society, the vast majority of people can be trusted?” Responses were recorded on a five-point Likert scale— “strongly disagree,” “disagree,” “neither agree nor disagree,” “agree,” and “strongly agree”—coded 1–5, with higher scores indicating greater social trust.

Social support was treated as a mediating variable. It was measured using the CGSS item: “During the past year, did you often meet with friends in your leisure time?” Responses were coded on a five-point scale—“never,” “several times a year or less,” “several times a month,” “several times a week,” and “every day”—assigned values of 1–5, respectively, with higher scores indicating greater social support. Because consistent multi-item support scales are unavailable across CGSS waves, we use contact frequency with friends as a proxy for support-related social connectedness and opportunities for support exchange. This proxy captures the interaction/contact facet of social support rather than its quality or type (e.g., emotional vs. instrumental) and does not cover family or formal support.

#### 2.2.4 Control variables

Prior research indicates that sociodemographic and socioeconomic factors shape individuals’ subjective well-being (Xiaoli et al., 2022; Qian et al., 2019; Xiaoli et al., 2019). To mitigate bias from omitted variables, we included individual-, household-, and region-level characteristics as control variables in the regression models. Individual characteristics comprise gender, age, age squared, household registration (hukou), religious belief, educational attainment, and annual personal income. Household characteristics include marital status, household income, household income rating, number of housing properties, and household risk investment status. Regional characteristics include GDP per capita and regional per capita disposable income.

Definitions and coding of all variables are provided in Appendix Table A1. Among the 53,967 sampled Chinese residents, subjective well-being ranges from 1 to 5, with a mean of 3.861, indicating a relatively high average level of happiness. Although the mean for watching sports events is below 0.5, the standard deviation is small, suggesting limited variation in the core explanatory variable. For the control variables, there are noticeable differences across indicators, and some exhibit variability, reflecting heterogeneity in individual, household, and regional characteristics among Chinese residents. These features provided a solid empirical basis for the subsequent analysis.

### 2.3 Data analysis

To examine the association between watching sports events and residents’ subjective well-being, we specify the following baseline regression model:


$$\mathrm{SW}B_{\mathrm{it}}=\beta_{0}+\beta_{1}\mathrm{WS}E_{\mathrm{it}}+\beta_{2}X_{\mathrm{it}}+\mu_{i}+\delta_{t}+\varepsilon_{\mathrm{it}}$$ (1)

Where i indexes provinces and t indexes survey years. The dependent variable $\mathrm{SW}B_{\mathrm{it}}$ denotes the subjective well-being of residents in province i and year t . The independent variable $\mathrm{WS}E_{\mathrm{it}}$ captures residents’ engagement in watching sports events. $X_{\mathrm{it}}$ is the vector of control variables. To account for time-invariant regional characteristics and common shocks in a given survey year, we include province dummy variables $\mu_{i}$ and survey-year dummy variables $\delta_{t}$ . Because the CGSS is a repeated cross-sectional survey and does not follow the same individuals over time, the model does not include individual fixed effects and should be interpreted as an OLS regression with province and year indicators. $\varepsilon_{\mathrm{it}}$ is the random disturbance term, and $\beta_{0}$ is the intercept.

Building on the foregoing theoretical mechanism, the positive association between watching sports events and residents’ subjective well-being may be statistically consistent with differences in social capital and health capital. To further investigate these potential pathways, we extend the baseline specification by adopting the two-step mediation testing framework of Jiang Ting (Jiang, 2022) and specify the following mediation models:


$$\mathrm{Me}d_{\mathrm{it}}=\beta_{0}+\beta_{1}\mathrm{WS}E_{\mathrm{it}}+\beta_{2}X_{\mathrm{it}}+\mu_{i}+\delta_{t}+\varepsilon_{\mathrm{it}}$$ (2)

Where $\mathrm{Me}d_{\mathrm{it}}$ denotes the mediating variables, including health capital (mental health and physical health) and social capital (social class, social trust, and social support). The definitions of the remaining variables are identical to those in Equation 1.

## 3 Results and analysis

### 3.1 Baseline regression

As shown in Table 2, Column (1) reports estimates from the OLS model with province and survey-year dummy variables and no additional controls, while Columns (2)–(4) sequentially add individual, household, and regional characteristics. Watching sports events was positively associated with residents’ subjective well-being in Column (1) (*β* = 0.0841, *p* < 0.01). After progressively introducing controls in Columns (2)–(4), the estimated coefficients declined (*β* = 0.0465, 0.0273, and 0.0268, respectively) but remained significant at the 1% level. This pattern suggests that the positive association persisted after accounting for a broad set of covariates. The gradual increase in R2 indicates enhanced explanatory power as additional controls were included. Although statistically significant, the magnitude is modest: *β* ≈ 0.02–0.03 on a 1–5 SWB scale corresponds to an average difference of roughly 0.5%–0.8% of the full scale range. Because sports-event viewing is a low-cost and widely practiced leisure behavior, even small average differences could matter at the population level, but the magnitude should not be overstated. Overall, these findings were consistent with Hypothesis H1.

**Table 2:** Baseline regression results.

Variables	SWB			
	(1)	(2)	(3)	(4)
WSE	0.0841^***^ (0.0000)	0.0465^***^ (0.0000)	0.0273^***^ (0.0017)	0.0268^***^ (0.0021)
Gender		−0.0813^***^ (0.0000)	−0.0454^***^ (0.0000)	−0.0454^***^ (0.0000)
Age		−0.0075 (0.4908)	−0.0237^**^ (0.0274)	−0.0233^**^ (0.0301)
Age^2^		0.0062^***^ (0.0000)	0.0058^***^ (0.0000)	0.0058^***^ (0.0000)
Household registration		0.0441^***^ (0.0000)	0.0344^***^ (0.0001)	0.0344^***^ (0.0001)
Religious belief		−0.0521^***^ (0.0000)	−0.0463^***^ (0.0001)	−0.0465^***^ (0.0001)
Educational attainment		0.0634^***^ (0.0000)	0.0380^***^ (0.0000)	0.0380^***^ (0.0000)
Annual personal income		0.1048^***^ (0.0000)	0.0057 (0.2338)	0.0057 (0.2362)
Marital status			0.1384^***^ (0.0000)	0.1383^***^ (0.0000)
Household income			0.0570^***^ (0.0000)	0.0570^***^ (0.0000)
Household income rating			0.2756^***^ (0.0000)	0.2757^***^ (0.0000)
Number of housing properties			0.0512^***^ (0.0000)	0.0512^***^ (0.0000)
Household risk investment status			0.0752^***^ (0.0000)	0.0750^***^ (0.0000)
GDP per capita				0.0164 (0.8238)
Regional per capita disposable income				0.1244 (0.3994)
Constant	3.8440^***^ (0.0000)	2.5919^***^ (0.0000)	2.0927^***^ (0.0000)	0.6614 (0.5710)
Province fixed effects	Yes			
Year fixed effects	Yes			
*N*	53,967			
adj. *R*^2^	0.0253	0.0508	0.1207	0.1207

SWB, subjective well-being; WSE, watching sports events; *p*-values in parentheses; ^*^p < 0.1, ^**^p < 0.05, ^***^p < 0.01.

### 3.2 Robustness checks

To ensure the reliability and robustness of the baseline regression results, we implemented four validation strategies: (1) altered the regression specification; (2) changed the estimation method for the dependent variable; (3) changed the estimation method for the independent variable; (4) expanded the set of control variables. Robustness checks are reported in Appendix Tables A2–A4 and yield consistent results.

First, we replaced the regression model. Using Ordered Logit, Ordered Probit, and Tobit models, we re-examined the association between watching sports events and residents’ subjective well-being. As shown in Appendix Table A2, the estimated coefficients on watching sports events were positive and statistically significant at the 1% level across all specifications, supporting the robustness of the preceding findings.

Second, we changed the estimation approach for the dependent variable. We recoded residents’ subjective well-being as a binary indicator, assigning 1 to “rather happy” and “very happy” and 0 otherwise, and then estimated an OLS model with province and survey-year dummy variables, as well as Logit and Probit models that include the same province and year indicators. As shown in Appendix Table A3, the coefficient on watching sports events was positive and statistically significant at the 10% level across all models, further supporting the robustness and reliability of the baseline results.

Third, we changed the estimation strategy for the explanatory variable. We treated watching sports events as a continuous measure, coding responses from “never” to “every day” as 1–5. We then estimated an OLS model with province and survey-year dummy variables, Ordered Logit, and Ordered Probit models; the results were reported in columns (1)–(3) of Appendix Table A4. Under this alternative coding, the coefficient on watching sports events was positive and statistically significant at the 10% level across all specifications, further supporting the reliability of the baseline results.

Fourth, we expanded the set of control variables. Specifically, we added ethnicity, BMI, health insurance participation, and homeownership status and re-estimated the OLS model; the results were reported in column (4) of Appendix Table A4. The coefficient on watching sports events remained positive and statistically significant at the 10% level, further supporting the reliability and robustness of the study’s findings.

### 3.3 Sensitivity analysis: propensity score matching (PSM)

To mitigate the influence of potential confounders and address biases arising from sample selection, we employed propensity score matching (PSM) as a sensitivity analysis to re-examine the baseline association between watching sports events and residents’ subjective well-being. Under the selection-on-observables assumption, PSM constructs, insofar as possible, a comparison group that is comparable to the focal group in the distribution of observable covariates.

This study primarily employed three matching procedures—nearest-neighbor (*k* = 2), radius (caliper = 0.01), and kernel—to match the treatment group (watching sports events) with the control group (not watching sports events). The post-matching diagnostics in Table 3 suggested a satisfactory balance, with no systematic differences between groups, thereby supporting the balancing assumption. As shown in Table 3, the estimated matched differences were 0.027 (nearest neighbor), 0.017 (radius), and 0.017 (kernel), each statistically significant at the 5% or 10% levels. These findings suggested that the positive association persisted after matching, consistent with Hypothesis H1, while not establishing causality.

**Table 3:** PSM results.

Matching methods	Watching sports events	Not watching sports events	ATT	S.E.	t
Nearest neighbor	3.928	3.901	0.027^**^	0.011	2.48
Radius matching	3.928	3.912	0.017^*^	0.009	1.78
Kernel matching	3.928	3.912	0.017^*^	0.009	1.80

Nearest-neighbor matching was implemented as 1-to-2 matching with replacement, and the radius (caliper) for radius matching was set to 0.01. Due to space constraints, the covariate-balance tables for the matched samples are not reported herein.

### 3.4 Heterogeneity tests

The baseline regression and robustness checks indicated that watching sports events was positively associated with residents’ subjective well-being. However, given China’s vast territory and a population of approximately 1.4 billion, it is necessary to investigate whether this association exhibits heterogeneity across groups. To further assess the association between watching sports events and residents’ subjective well-being, this section examines heterogeneity by region, gender, and educational attainment.

#### 3.4.1 Regional heterogeneity

As shown in Table 4, the association between watching sports events and residents’ subjective well-being was statistically significant in the eastern, central, and western regions: the central region was significant at the 1% level, whereas the eastern and western regions were significant at the 10% level. The regression coefficients further suggested that the well-being gains associated with watching sports events were larger in the west than in the east (0.0319 > 0.0206). By contrast, the association for the northeast was not statistically significant.

**Table 4:** Heterogeneity analysis by region.

Variables	SWB			
	Eastern region	Central region	Western region	Northeast region
	(1)	(2)	(3)	(4)
WSE	0.0206^*^ (0.0987)	0.0608^***^ (0.0026)	0.0319^*^ (0.0919)	0.0024 (0.9227)
Constant	13.9807^***^ (0.0000)	9.3148^**^ (0.0256)	3.8074 (0.1730)	−23.1961 (0.3999)
Province fixed effects	Yes			
Year fixed effects	Yes			
Control variables	Yes			
*N*	20,735	12,498	13,775	6,959
adj. *R*^2^	0.1027	0.1333	0.1265	0.1216

SWB, subjective well-being; WSE, watching sports events; *p*-values in parentheses; ^*^p < 0.1, ^**^p < 0.05, ^***^p < 0.01.

These patterns indicate regional heterogeneity in the association between watching sports events and residents’ subjective well-being, providing support for Hypothesis H4a. Potential explanations are discussed in the Discussion section.

#### 3.4.2 Gender heterogeneity

As shown in Table 5, the association between watching sports events and residents’ subjective well-being was statistically significant for men (*p* < 0.01), whereas the corresponding association for women was not significant. Accordingly, Hypothesis H4b was supported.

**Table 5:** Heterogeneity analysis by gender.

Variables	SWB	
	Male	Female
	(1)	(2)
WSE	0.0348^***^ (0.0025)	0.0187 (0.1664)
Constant	0.5355 (0.7493)	0.5688 (0.7301)
Province fixed effects	Yes	
Year fixed effects	Yes	
Control variables	Yes	
*N*	28,370	25,596
adj. *R*^2^	0.1260	0.1166

SWB, subjective well-being; WSE, watching sports events; *p*-values in parentheses; ^*^p < 0.1, ^**^p < 0.05, ^***^p < 0.01.

#### 3.4.3 Educational heterogeneity

As shown in Table 6, the association between watching sports events and residents’ subjective well-being was significant for respondents with a high school education (*p* < 0.05) and for those with a college education or above (*p* < 0.10), whereas the corresponding association was not significant among respondents with primary or junior-high education. This pattern was consistent with Hypothesis H4c.

**Table 6:** Regional heterogeneity test results.

Variables	SWB			
	Primary school or below	Junior high	High school	University or above
	(1)	(2)	(3)	(4)
WSE	0.0254 (0.2882)	0.0096 (0.5695)	0.0342^**^ (0.0459)	0.0257^*^ (0.0757)
Constant	−1.4521 (0.5640)	1.9234 (0.3679)	0.9268 (0.7252)	6.3378^***^ (0.0041)
Province fixed effects	Yes			
Year fixed effects	Yes			
Control variables	Yes			
*N*	18,194	15,900	10,249	9,622
adj. *R*^2^	0.1508	0.1082	0.1017	0.0710

SWB, subjective well-being; WSE, watching sports events; *p*-values in parentheses; ^*^p < 0.1, ^**^p < 0.05, ^***^p < 0.01.

### 3.5 Mediation model results

The foregoing analyses supported a positive association between watching sports events and subjective well-being across multiple robustness checks. Building on the theoretical framework, watching sports events may relate to residents’ subjective well-being through health capital and social capital. Following Jiang’s (2022) approach, we employed a two-step mediation model to examine these potential pathways, as specified in Equation 2. Given the repeated cross-sectional design, this mechanism analysis should be interpreted as “statistical mediation” (exploratory pathway-consistent evidence) rather than causal mediation. Given theoretically plausible links between the mediators and subjective well-being, we focused on whether watching sports events was associated with each mediator. The mediation results are presented in Table 7. As indicated by Column (1), consistent with the baseline regressions, watching sports events was positively associated with residents’ subjective well-being; the mediator patterns are summarized below.

**Table 7:** Mediation effects of watching sports events on residents’ subjective well-being.

Variables	SWB	Mental health	Physical health	Social class	Social trust	Social support
	(1)	(2)	(3)	(4)	(5)	(6)
WSE	0.0268^***^ (0.0021)	−0.0087 (0.4091)	0.0382^***^ (0.0002)	0.1457^***^ (0.0000)	0.0377^***^ (0.0011)	0.2437^***^ (0.0000)
Constant	0.6614 (0.5710)	−3.5781^**^ (0.0143)	−2.8320^**^ (0.0486)	−0.3030 (0.9000)	1.6972 (0.2583)	3.9099^***^ (0.0030)
Province fixed effects	Yes					
Year fixed effects	Yes					
Control variables	Yes					
*N*	5,3,967					
adj. *R*^2^	0.1207	0.0967	0.2180	0.2347	0.0429	0.1557

SWB, subjective well-being; WSE, watching sports events; *p*-values in parentheses; ^*^p < 0.1, ^**^p < 0.05, ^***^p < 0.01.

Column (2) of Table 7 indicates that the association between watching sports events and mental health was not statistically significant; thus, Hypothesis H2a was not supported. A plausible explanation is that any short-lived pleasure from wins may be offset by stronger stress responses following losses (e.g., elevated cortisol) (Tsuji et al., 2021), so that watching sports events does not necessarily improve spectators’ mental health (Newson et al., 2020).

Column (3) shows that the coefficient for watching sports events was 0.0382 and was statistically significant at the 1% level; therefore, watching sports events was positively associated with residents’ physical health. This pattern is consistent with the view that spectatorship may be associated with greater exercise intention and healthier lifestyles (Watanabe et al., 2020; Yargic and Kurklu, 2020), and physical health is a key correlate of residents’ subjective well-being (Mathentamo et al., 2024; Wang et al., 2022). Accordingly, the results were consistent with the view that physical health may statistically account for part of the association between watching sports events and subjective well-being; thus, Hypothesis H2b was supported.

Column (4) shows that the coefficient for watching sports events was 0.1457 and was statistically significant at the 1% level; therefore, watching sports events was positively associated with residents’ perceived social class. Although viewing itself does not directly change socioeconomic position, sport-related engagement may expand social ties and symbolic resources that shape class identification (Harris and Parker, 2009; Spaaij, 2009). Building on prior research, perceived social class is an important determinant of residents’ subjective well-being (Varghese et al., 2022; Yeh et al., 2015) through reduced resource scarcity and enhanced self-identification (Li et al., 2024; Chen et al., 2021). Accordingly, the results were consistent with the view that perceived social class may statistically account for part of the association between watching sports events and subjective well-being; thus, Hypothesis H3a was supported.

Column (5) shows that the coefficient for watching sports events was 0.0377 and was statistically significant at the 1% level; therefore, watching sports events was positively associated with residents’ social trust. Sports events can foster shared emotions and collective identity and transmit prosocial values (e.g., fair play and teamwork) (Lee et al., 2016; Zhou et al., 2021), and social trust is positively associated with subjective well-being (Helliwell et al., 2016). Accordingly, the results were consistent with the view that social trust may statistically account for part of the association between watching sports events and subjective well-being; thus, Hypothesis H3b was supported.

Column (6) indicates that the coefficient for watching sports events was 0.2437 and was statistically significant at the 1% level; thus, watching sports events was positively associated with residents’ level of social support. Spectatorship often occurs in shared settings and may strengthen social interaction and emotional bonds (Guo et al., 2024; Stieler and Germelmann, 2016), and social support is a robust correlate of subjective well-being by buffering stress and enhancing positive affect (Anisa et al., 2024; Zhang et al., 2024). Accordingly, the results were consistent with the view that social support may statistically account for part of the association between watching sports events and subjective well-being; thus, Hypothesis H3c was supported.

## 4 Discussion

Drawing on eight waves of CGSS data (2010–2023), we examined whether routine sports-event watching is associated with residents’ subjective well-being and whether health capital and social capital are statistically consistent with potential pathways. Below, we interpret the main findings and discuss their implications.

### 4.1 The impact of watching sports events on subjective well-being

Routine sports-event watching was positively associated with residents’ subjective well-being, broadly consistent with prior findings (Guo et al., 2024; Ramchandani et al., 2022; Kinoshita et al., 2024a). As a core modality of the sports industry, sports-event viewing can generate value by facilitating social interaction (Madrigal, 2003), enhancing public well-being (Kinoshita et al., 2024b), and supporting the coordinated development of related sectors. Competitive sport embodies fair play, respect for rules, teamwork, and persistence. When spectators resonate with these values (Lumpkin, 2008), they may derive a sense of meaning and social identification (Lee et al., 2016), which may be linked to higher life satisfaction and positive affect (Guo et al., 2024). In addition, watching sports events provides short-term immersion that may help buffer the effects of work and family pressures on well-being (Yoshida et al., 2023); existing research suggests that this association may be especially pronounced among people with limited time and energy (Lin et al., 2023).

At the same time, routine sports viewing may also involve theoretically relevant countervailing mechanisms. For example, viewing often entails prolonged sedentary time and extended screen exposure, which are linked to adverse cardiovascular and metabolic outcomes in public-health guidelines and observational evidence on sedentary behavior and television viewing (Sun et al., 2015). In addition, major competitions are frequently scheduled at night (including cross-time-zone broadcasts), and late-night viewing can disrupt sleep timing and recovery, which may undermine next-day mood and well-being (Jackowska et al., 2024). Viewing occasions may also be accompanied by unhealthy co-consumption (e.g., energy-dense snacks) or alcohol use in some contexts, which could attenuate net health benefits (Koenigstorfer, 2018). These offsetting processes imply that the overall association between routine viewing and SWB may be positive on average while still being heterogeneous across viewing patterns (e.g., excessive duration, night-time viewing, or unhealthy co-consumption).

Moreover, alternative explanations cannot be ruled out. For example, individuals with higher baseline well-being may be more inclined to watch sports events (reverse causality), and unobserved factors (e.g., personality, leisure preference, local media exposure, or community environment) may jointly influence both spectating and well-being.

### 4.2 Heterogeneity in subjective well-being

We observed regional differences in the association between sports-event watching and subjective well-being. In the central region, economic restructuring and rising disposable income may have shifted consumption toward leisure. Policies promoting an event economy in second- and third-tier cities may also increase residents’ exposure to sports events and related well-being gains. In western China, the growth of grassroots tournaments (e.g., “Village BA” and the “Village Super League”) and the diffusion of short-video/live-streaming platforms (Biao, 2024) may have expanded viewing opportunities. However, weaker transport accessibility, lower population density, and gaps in public sports services may constrain the overall association (Zhang et al., 2023). In the eastern region, sports-event supply and related services are relatively saturated, and abundant leisure alternatives may dilute the marginal association of spectating (Wang et al., 2024). In the northeastern region, industrial stagnation and population outflow may constrain both event supply and participation, thereby weakening the association. Overall, these subgroup patterns are descriptive and should not be interpreted as differential causal effects, given potential multiple-testing concerns and unobserved subgroup-specific confounding.

We found gender differences in the association between sports-event watching and subjective well-being. The association was evident among men but not among women, consistent with prior findings (Hirayama et al., 2023). One explanation is that men may be more likely to engage as “fans,” showing higher involvement and stronger affective responses; stronger fan identification has been linked to higher subjective well-being (Gantz and Wenner, 1991). Experimental evidence also indicates that men display higher electrodermal responses when watching team sports, while women’s physiological responses are smaller or nonsignificant (Hosťovecký et al., 2022) which may imply weaker emotional activation during spectating. Differences in viewing contexts, social roles, and reporting styles may also contribute to these patterns. The balance between benefits (identity and social sharing) and costs (stress or sleep disruption) may differ by gendered viewing contexts, which cannot be directly tested with the available measures.

We also observed educational differences in the association between sports-event watching and subjective well-being. Respondents with a high school education or college and above may have greater access to information and resources for viewing, and they may be more willing to discuss competitions, which could amplify the social and emotional benefits of spectating (Thrane, 2001). Prior work also links sports spectatorship and fan engagement to life satisfaction and well-being (Inoue et al., 2017b). The stronger association among those with a high school education may reflect salient social needs that are met through shared sports conversations. Among respondents with college or higher education, academic and work pressures may reduce the marginal benefit of viewing, yielding a smaller association than that observed for the high school group. By contrast, respondents with primary or junior-high education may watch less frequently due to constraints in information channels, economic conditions, or interest; as a result, any happiness gains may be diluted. Future research should examine whether differences in health literacy and time use moderate the potential downsides of viewing (e.g., sedentary time or late-night viewing).

### 4.3 The mechanism of watching sports events affecting subjective well-being

Mediation analyses were consistent with social capital—social class, social trust, and social support—statistically accounting for part of the association between sports-event watching and subjective well-being, in line with related research (Guo et al., 2024; Lin, 2022). Sports spectatorship may broaden social networks and increase interaction across social strata, generating “cross-class” ties that may promote social integration and expand life opportunities (Rungo and Sánchez-Santos, 2022). As a collective activity, watching can create shared emotional experiences and common conversational themes (Yoshida et al., 2023), and it is often undertaken with family members, friends, or like-minded peers. Consistent with collective emotion theory, the ties formed through shared interests and feelings may enhance interpersonal and group trust and strengthen individuals’ connections to society (Wann et al., 2008). Supporting the same team or athlete can also foster belonging and identification, which may be associated with higher subjective well-being. Given the repeated cross-sectional design, these mediation results should be interpreted as correlational pathways rather than confirmed causal mechanisms.

Mediation analyses were also consistent with physical health as a statistical pathway linking sports-event watching and subjective well-being (Ramchandani et al., 2022; Kawakami et al., 2024). Under some conditions, watching sports events can motivate participation in physical exercise (Teare and Taks, 2021). For example, strong performances by athletes or teams may increase fans’ intentions to exercise and their actual activity, translating spectating into a more active lifestyle (Watanabe et al., 2020). Better physical health is a well-established correlate of higher subjective well-being (Wang et al., 2022) and may contribute through reduced disease burden, greater vitality, and improved emotional states (Iwon et al., 2021). However, viewing is often sedentary; public-health guidance emphasizes limiting sedentary time and replacing it with physical activity (Bull et al., 2020). Thus, the net association may depend on whether watching complements active lifestyles (e.g., motivating exercise) or substitutes for them (e.g., prolonged sitting). Future studies should incorporate more granular time-use and viewing-context measures (duration, time of day, co-viewing, and concurrent activity) to test these competing pathways directly.

In contrast, the pathway through mental health was not statistically significant. One possibility is that live or high-stakes matches heighten stress reactivity (e.g., increased salivary cortisol), especially after defeats, which may offset any average improvement in mental well-being (Newson et al., 2020). Related evidence shows spikes in acute cardiovascular events during emotionally intense competitions (e.g., the 2006 FIFA World Cup) (Wilbert-Lampen et al., 2008), highlighting that spectating can entail physiological and psychological stress and thus may not necessarily improve mental health. Taken together, our findings support a nuanced interpretation: routine viewing is positively associated with subjective well-being on average, partly through social-capital-related resources and (in our data) physical health, yet specific viewing patterns or competitive contexts may simultaneously generate stress, sleep disruption, and sedentary exposure that attenuate benefits for some individuals.

## 5 Conclusion

Using eight waves of CGSS data (2010–2023), we examined the association between watching sports events and residents’ subjective well-being and explored whether health capital and social capital were statistically consistent with potential pathways. Across OLS models with province and survey-year dummy variables, robustness checks, heterogeneity analyses, and PSM sensitivity analyses, watching sports events was positively associated with subjective well-being. Because the CGSS provides repeated cross-sectional data, all findings should be interpreted as associations rather than causal effects. The study yields three principal conclusions:

First, watching sports events was positively associated with residents’ subjective well-being. This conclusion remained robust after a series of checks, including alternative specifications of the independent variable, alternative measures of the dependent variable, changes to the regression model, the inclusion of additional control variables, and propensity score matching (PSM). At the same time, routine viewing may entail countervailing mechanisms (e.g., prolonged sedentary time, late-night viewing, or stress responses during high-stakes matches) that could attenuate benefits for some individuals, implying that the net association may depend on viewing patterns and contexts.

Second, the association between watching sports events and residents’ subjective well-being varied across subgroups. Regional heterogeneity tests suggested that the well-being gains associated with watching sports events were greatest in the western region, followed by the central and eastern regions, whereas the association was not evident in the northeast. By gender, the association was statistically significant for men but not for women. By education, the association was most pronounced among residents with a high school education, followed by those with college or higher education; for residents with only primary or junior-high education, the association was not statistically significant.

Third, the mediation analysis suggested that social capital—comprising social class, social trust, and social support—statistically accounted for part of the association between watching sports events and residents’ subjective well-being. Within health capital, physical health also statistically accounted for part of the association between watching sports events and subjective well-being. By contrast, the mediating role of mental health was not statistically significant, indicating that in our measurement and model specification, mental health did not statistically account for the association; this null finding may reflect offsetting affective processes during viewing or the limited scope of the available mental-health measure. Overall, these results support a cautious and balanced interpretation: routine sports viewing is positively associated with SWB on average, but promoting “healthy viewing” (e.g., avoiding excessive duration and maintaining active breaks) is important when translating findings into practice.

## 6 Implications

Based on the results and discussion, we propose four recommendations:

First, broaden access to spectating by expanding broadcast coverage and supporting a diversified supply of sporting events. Regions showing stronger associations (e.g., the central and western regions) may prioritize event quality and accessible viewing opportunities, while areas with weaker associations (e.g., the northeast) may focus on strengthening local sports culture and targeted promotion.

Second, enhance the social benefits of spectating by using sporting events as platforms for community connection—for example, organizing group-viewing activities or public viewing spaces to facilitate interaction, belonging, and social support.

Third, link spectating with health promotion by pairing major competitions with community exercise programs or event-themed fitness challenges, encouraging spectators to translate viewing motivation into physical activity.

Fourth, improve inclusiveness and accessibility through tailored outreach (e.g., diversified content and dissemination for women; plain-language commentary and sports-culture outreach for lower-education groups) to lower participation barriers and broaden engagement.

## 7 Limitations

Although the findings of this study have theoretical and practical significance, several limitations warrant note.

First, constrained by the CGSS questionnaire design, watching sports events was measured using a single indicator (spectating frequency). Future targeted surveys could incorporate viewing duration, timing, and modes/platforms to better characterize routine viewing.

Second, key constructs (SWB and the proposed mechanism variables) were measured using single-item indicators in the CGSS, and some mediators were proxied by facet-specific items. These measurement constraints may introduce error and limit construct coverage; therefore, the mechanism results should be interpreted as indicative rather than definitive. Future work should employ validated multi-item scales and richer measurement batteries when feasible.

Third, the analyses were based on repeated cross-sectional data, which limits causal interpretation. Temporal ordering cannot be established, and reverse causality and residual confounding cannot be ruled out. Accordingly, longitudinal or quasi-experimental designs are needed to more rigorously assess causal pathways.

Fourth, the CGSS data were available only at the provincial level, limiting finer-grained contextual analyses of event supply and media access. Future research could link survey data with city-level or geocoded contextual measures.

## Data Availability

Publicly available datasets were analyzed in this study. This data can be found at: http://cgss.ruc.edu.cn/.
