# Supplementary material for: Watching sports events and residents’ subjective well-being: evidence from the CGSS and the potential roles of health and social capital
Source: Front Psychol. 2026 Feb 4;17:1775253. doi: 10.3389/fpsyg.2026.1775253 (PMC12913550; doi:10.3389/fpsyg.2026.1775253)
Supplement: Supplementary file 1 [file Table_1.DOCX]

**TABLE A1** Variable definitions and descriptive statistics (N=53,967).

| **Types** | | **Variables** | **Definition** | **mean** | **sd** | **min** | **max** |
| --- | --- | --- | --- | --- | --- | --- | --- |
| Dependent variable | | Subjective Well-being (SWB) | 1 = Very unhappy,  2 = Relatively unhappy,  3 = Neither happy nor unhappy,  4 = Relatively happy,  5 = Very happy | 3.861 | 0.825 | 1 | 5 |
| Independent variable | | Watching Sports Events (WSE) | 1 = Yes; 0 = No | 0.200 | 0.400 | 0 | 1 |
| Mediator variables | Health capital | Mental health | 1 = Always,  2 = Often,  3 = Sometimes,  4 = Seldom,  5 = Never | 3.902 | 0.976 | 1 | 5 |
|  |  | Physical health | 1 = Very unhealthy, 2= Relatively unhealthy,  3 = Average,  4 = Relatively healthy,  5 = Very healthy | 3.574 | 1.072 | 1 | 5 |
|  | Social capital | Social class | Scale: 1–10 (10 = highest; 1 = lowest). | 4.258 | 1.703 | 1 | 10 |
|  |  | Social trust | 1 = Strongly disagree,  2 = Disagree,  3 = Neither agree nor disagree,  4 = Agree,  5 = Strongly agree | 3.494 | 1.012 | 1 | 5 |
|  |  | Social support | 1 = Never,  2 = Several times a year or less,  3 = Several times a month,  4 = Several times a week,  5 = Every day | 2.355 | 0.970 | 1 | 5 |
| Control variables | Individual characteristics | Gender | 1 = Male, 0 = Female | 0.526 | 0.499 | 0 | 1 |
|  |  | Age | 1= Youth (18-34 years old),  2 = Middle-aged adults (35-64 years old),  3 = Elderly (≥65 years old) | 2.050 | 0.628 | 1 | 3 |
|  |  | Age^2 | Age^2/100 | 28.55 | 16.44 | 4.410 | 72.25 |
|  |  | Household registration | 1 = Rural, 0 = Urban | 0.526 | 0.499 | 0 | 1 |
|  |  | Religious belief | 1 = No, 0 = Yes | 0.893 | 0.309 | 0 | 1 |
|  |  | Educational attainment | 1 = Primary school or below,  2 = junior high,  3 = high school,  4 = University or above | 2.209 | 1.094 | 1 | 4 |
|  |  | Annual personal income | ln(annual personal income) | 9.781 | 1.266 | 6.492 | 12.61 |
|  | Household characteristics | Marital status | 1 = Married,  0 = Unmarried and other | 0.798 | 0.402 | 0 | 1 |
|  |  | Household income | ln(household income) | 10.57 | 1.158 | 7.189 | 13.12 |
|  |  | Household income rating | 1 = Far below average,  2 = Below average,  3 = Average,  4 = Above average,  5 = Far above average | 2.627 | 0.732 | 1 | 5 |
|  |  | Number of housing properties | Number of household properties | 1.117 | 0.608 | 0 | 10 |
|  |  | Household risk investment status | 1 = No, 0 = Yes | 0.772 | 0.420 | 0 | 1 |
|  | Regional characteristics | GDP per capita | ln(GDP per capita) | 10.89 | 0.510 | 9.849 | 12.14 |
|  |  | Regional per capita disposable income | ln(regional per capita disposable income) | 10.07 | 0.476 | 9.150 | 11.23 |

**TABLE A2** Robustness checks: alternative regression models.

| **Variables** | **SWB** | | | | | |
| --- | --- | --- | --- | --- | --- | --- |
|  | **Ordered Logit model** | | **Ordered Probit model** | | **Tobit model** | |
|  | **(1)** | **(2)** | **(3)** | **(4)** | **(5)** | **(6)** |
| WSE | 0.1715^***^  (0.0000) | 0.0654^***^  (0.0044) | 0.1033^***^  (0.0000) | 0.0382^***^  (0.0031) | 0.0926^***^  (0.0000) | 0.0321^***^  (0.0025) |
| Constant |  |  |  |  | 3.9132^***^  (0.0000) | -0.2317  (0.8722) |
| Province fixed effects | Yes | | | | | |
| Year fixed effects | Yes | | | | | |
| Control variables | No | Yes | No | Yes | No | Yes |
| *N* | 53,967 | | | | | |
| Pseudo *R*^2^ | 0.0136 | 0.0560 | 0.0128 | 0.0555 | 0.0104 | 0.0464 |

SWB, Subjective well-being; WSE, Watching Sports Events; *p*-values in parentheses; ^*^*p* < 0.1, ^**^*p* < 0.05, ^***^*p* < 0.01.

**TABLE A3** Robustness checks: alternative estimation methods for the dependent variable.

| **Variables** | **SWB (binary variable)** | | |
| --- | --- | --- | --- |
|  | **OLS model** | **Logit model** | **Probit model** |
|  | **(1)** | **(2)** | **(3)** |
| WSE | 0.0088^*^  (0.0520) | 0.0601^*^  (0.0502) | 0.0320^*^  (0.0676) |
| Constant | 0.2092  (0.7221) | -1.4909  (0.6965) | -1.0656  (0.6281) |
| Province fixed effects | Yes | | |
| Year fixed effects | Yes | | |
| Control variables | Yes | | |
| *N* | 53,967 | | |
| adj. *R*^2^/Pseudo *R*^2^ | 0.0973 | 0.0955 | 0.0946 |

SWB, Subjective well-being; WSE, Watching Sports Events; *p*-values in parentheses; ^*^*p* < 0.1, ^**^*p* < 0.05, ^***^*p* < 0.01.

**TABLE A4** Robustness checks: alternative estimation methods for the independent variable and additional controls.

| **Variables** | **SWB** | | | |
| --- | --- | --- | --- | --- |
|  | **OLS model** | **Ordered Logit model** | **Ordered Probit model** | **Additional controls** |
|  | **(1)** | **(2)** | **(3)** | **(4)** |
| WSE (continuous variable) | 0.0147^***^  (0.0034) | 0.0424^***^  (0.0013) | 0.0231^***^  (0.0017) |  |
| WSE (binary variable) |  |  |  | 0.0244^***^  (0.0064) |
| Constant | 0.6685  (0.5670) |  |  | 1.4329  (0.2804) |
| Province fixed effects | Yes | | | |
| Year fixed effects | Yes | | | |
| Control variables | Yes | | | |
| *N* | 53,967 | 53,967 | 53,967 | 51,112 |
| adj. *R*^2^/Pseudo *R*^2^ | 0.1207 | 0.0560 | 0.0555 | 0.1229 |

SWB, Subjective well-being; WSE, Watching Sports Events; *p*-values in parentheses; ^*^*p* < 0.1, ^**^*p* < 0.05, ^***^*p* < 0.01.
